# A Profile of the SAIL Databank on the UK Secure Research Platform

**DOI:** 10.23889/ijpds.v4i2.1134

**Published:** 2019-11-20

**Authors:** KH Jones, DV Ford, S Thompson, RA Lyons

**Affiliations:** 1 Population Data Science, Swansea University Medical School, Singleton Park, Swansea SA2 8PP

## Abstract

**Background:**

The Secure Anonymised Information Linkage (SAIL) Databank is a national data safe haven of de identified datasets principally about the population of Wales, made available in anonymised form to researchers across the world. It was established to enable the vast arrays of data collected about individuals in the course of health and other public service delivery to be made available to answer important questions that could not otherwise be addressed without prohibitive effort. The SAIL Databank is the bedrock of other funded centres relying on the data for research.

**Approach:**

SAIL is a data repository surrounded by a suite of physical, technical and procedural control measures embodying a proportionate privacy-by-design governance model, informed by public engagement, to safeguard the data and facilitate data utility. SAIL operates on the UK Secure Research Platform (SeRP), which is a customisable technology and analysis platform. Researchers access anonymised data via this secure research environment, from which results can be released following scrutiny for disclosure risk. SAIL data are being used in multiple research areas to evaluate the impact of health and social exposures and policy interventions.

**Discussion:**

Lessons learned and their applications include: managing evolving legislative and regulatory requirements; employing multiple, tiered security mechanisms; working hard to increase analytical capacity efficiency; and developing a multi-faceted programme of public engagement. Further work includes: incorporating new data types; enabling alternative means of data access; and developing further efficiencies across our operations.

**Conclusion:**

SAIL represents an ongoing programme of work to develop and maintain an extensive, whole population data resource for research. Its privacy-by-design model and UK SeRP technology have received international acclaim, and we continually endeavour to demonstrate trustworthiness to support data provider assurance and public acceptability in data use. We strive for further improvement and continue a mutual learning process with our contemporaries in this rapidly developing field

## Introduction

The Secure Anonymised Information Linkage (SAIL) Databank was established by the Population Data Science group at Swansea University (Wales, UK) in 2007 with core funding from Health and Care Research Wales (HCRW) of the Welsh Government. SAIL was created in recognition of the immense, untapped research potential of individual-level data collected in the course of health and other public service delivery. Its predicate was that making these vast arrays of data accessible safely would open up ways to answer important questions that could not otherwise be addressed without prohibitive effort and cost. SAIL is the bedrock of further Population Data Science investments hosted at Swansea University, including the Economic & Social Research Council funded Administrative Data Research Wales [1] and the Medical Research Council funded Health Data Research UK collaboration between Swansea University and Queen’s University Belfast (Northern Ireland, UK) [2]. Through these and other developments, the scope of SAIL data has expanded to include administrative data that were not previously accessible (such as education, housing and employment) and emerging health data types (such as genomic, free-text and imaging). In this way, SAIL is an increasingly rich resource for population data science – *‘the science of data about people’*, including wider factors that influence wellbeing [3]. This accords with the need for a better understanding of *‘the complex array of interlinking factors that influence the health of the public’*, identified by the UK Academy of Medical Sciences [4]. These issues transcend traditional disciplinary, sectoral and geographical boundaries and require integrating aspects of natural, social and health sciences, an aspiration to shift focus to prevention and early intervention to optimise resource utilisation and achieve population impact.

The aim of this paper is to present a profile of the SAIL Databank including its population setting, operating model, underpinning architecture and technology, data governance, data linkage, data sources, how data access is enabled and examples of noteworthy outputs.

## Approach

### Population setting

The SAIL Databank is a national data safe haven of de identified datasets about the population of Wales, made available in anonymised form for research. Wales is a principality in the United Kingdom with a population of 3.18 million in 2018 [5]. The main urban centres in terms of population numbers are the capital Cardiff, followed by Swansea and Newport across south and west Wales, and Wrexham in the north. In terms of area, the majority of Wales is rural, particularly non-industrialised mid-Wales and where much of the land is agricultural. SAIL data encompasses the population of Wales, and because the data are longitudinal, it includes records of over 5 million people who have been recipients of public services in Wales.

### Operating model

SAIL data are housed in a data repository surrounded by a suite of physical, technical and procedural control measures, which taken together comprise a privacy-by-design, proportionate governance model [6,7]. This will be described more fully below. Here we describe the reasoning for choosing to establish SAIL as a data repository and the principles that guide its operation.

In seeking to establish a system for making population data available for research in anonymised form, there were numerous factors to be considered. We consulted widely with government, regulatory and professional agencies, and we sought to learn from existing systems, mainly in other parts of the world with long-established data centres [8-10]. The main factors that guided our decision to create a repository, rather than a distributed model with federated data access, were largely pragmatic i.e. they were a combination of problem solving, gaining the advantage of available opportunities, minimising the demand on data providers and maximising efficiency.

Through discussions with stakeholders, we concluded that many public sector IT systems were not sufficiently stable to cope with data access and processing at source. Coupled with this, we were able to develop technical processes to minimise the demand on data providers transferring their data to SAIL. We had the advantages of high performance computing capacity at Swansea University without which the initial cost of set up would have been prohibitive, and the availability of a National Health Service based Trusted Third Party (TTP) in the NHS Wales Informatics Service (NWIS) to process identifiable data for the data provider. As well as enacting the separation principle by which SAIL does not handle person-identifiable data (PID), NWIS maintains the Welsh Demographic Service (WDS) database that acts as a proxy for a Wales population register, and against which records can be reliably matched. Further drivers for a repository model were the ability to monitor data quality and completeness, and to apply proportionate controls on data access. We were able to devise a model that enables SAIL to hold data in accordance with UK data protection legislation [11,12], meeting with regulatory requirements and those of data provider due diligence [6,7]. Importantly, using a repository model meant that all the data were in place and readily available for research use, subject to approvals.

### Architecture and information technology

**Figure 1: The SAIL Secure Research Platform:**
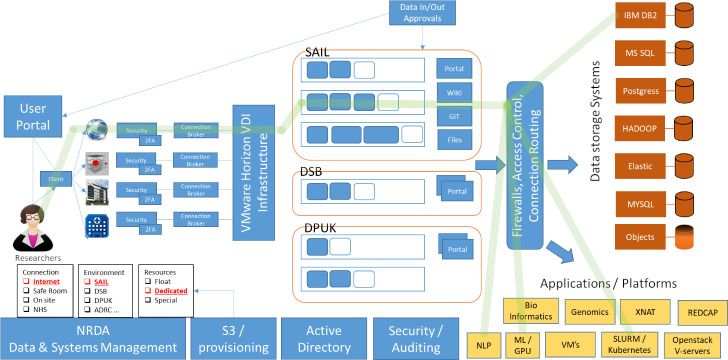
SAIL operates on a secure research platform (UK SeRP). Beginning at the left of the diagram, wherever researchers are based, they access data through a provisioned, secure, research ready desktop using VMware Horizon infrastructure. The connection from the user’s terminal to the desktop is strongly encrypted and access control prevents data being transferred outside the desktop environment. The end user is authenticated through both user credentials and two factor authentication tokens. Provisioned desktops come in a variety of capacities and configurations to suit the type of analysis that the end user and project needs. As part of the research environment there are shared project spaces to enable collaboration through database space, file store, wiki, Git (source control) as well as access to wider support and help materials. UK SeRP has many shared infrastructure components that can help deliver the programme’s objectives or specific project needs. SAIL uses IBM DB2 as its data warehouse due to the massively parallel processing (MPP) architecture and the ability to scale to suit the needs of such a large repository and the big data needs that this drives. To support specific project needs, other UK SeRP components can be made available, such as the HPC cluster or Kubernetes cluster to support processing pipelines, or GPU and AI cluster for training computing models. Through the provision of virtual machines or container environment, SAIL can support more complex methodological developments that require bespoke infrastructure to support development or deployment of tailored solutions. Business intelligence tools such as Tableau, R Shiny and PowerBI (not shown) are also available. Two other UK SeRP instances (Data Science Building projects (DSB) and Dementias Platform UK (DPUK)) are included on the diagram to help illustrate the customisability of the platform, since these will operate using different components, or other governance regimens to SAIL.

**Figure 2: The National Research Data Appliance:**
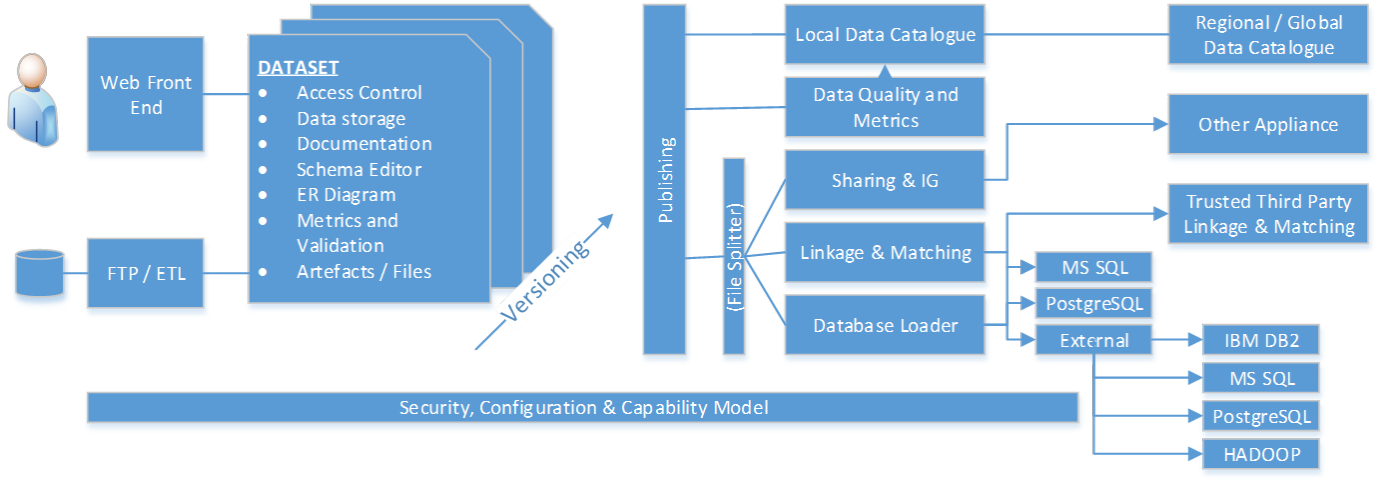
The various components of the National Research Data Appliance (NRDA) are shown. The entire UK SeRP environment is controlled by “Security 3 (S3)”, a feature of the National Research Data Appliance (NRDA) allowing the tenancy to be managed and controlled by non-technical team members. The user accounts and projects are defined and managed through a user interface (shown on the left) allowing different levels of access, even allowing project PI to self-manage project membership. (This self-management feature is not enabled for SAIL.) This system allows the infrastructure configuration, project configuration and governance structure to be documented, and all system components orchestrated, through the user interface. Which parts of the infrastructure are accessible and which projects within that environment are enacted in the particular tenancy model. The S3 model is periodically checked against the infrastructure and any nonconformity is corrected and reported.

SAIL is hosted, managed and provisioned within a tenancy on the UK Secure Research Platform (SeRP). UK SeRP is an ISO27001 approved customisable technology and analysis platform with a range of functionalities, developed by the SAIL technical team [13]. We use the term tenancy to refer to the particular specifications and services required, including software preferences and data governance model, of a client wishing to use the infrastructure. UK SeRP enables multiple, complex datasets to be managed, analysed and shared in accordance with data provider permissions and jurisdictional legislative and regulatory requirements (Figure 1). The decisions on which tools are implemented for a particular tenancy is made between the tenant and UK SeRP taking into account the requirements of data providers and the regulatory status of the provision of datasets. Each instance of UK SeRP is controlled by a National Research Data Appliance (NRDA, Figure 2), a set of modules to provide dataset management, access control, infrastructure management and governance model implementation. NRDA is the control management system, while UK SeRP is the secure analytic environment for users to access and conduct research.

### Governance, legislation and management

SAIL is classed as an Infrastructure Support Service in the terms of its funding from HCRW. This means SAIL has a fundamental role in enabling research to take place and in providing a specialist service to research bodies across Wales and beyond. SAIL is hosted by Swansea University and sits under its auspices for organisational accountability. Strategic direction is provided by the SAIL Management Group with guidance from an International Scientific Advisory Committee, and day to day running is managed by an Operations Group.

The main data protection legislation and regulations with which SAIL needs to comply are the EU General Data Protection Regulation [11], the UK Data Protection Act [12] and the Common Law Duty of Confidentiality (CLDC) [14]. The UK DPA is read alongside the GDPR to enact the GDPR in the UK. The CLDC applies in a relationship (such as a doctor/patient consultation) where information is understood to be given in confidence, and is based on precedent and case law, rather than being embodied in a statutory document. The GDPR sets out conditions for the lawful processing of PID, both general data (such as name) and special categories of data (such as health records). Anyone processing PID must be able to justify their lawful basis for doing so under the GDPR.

As noted above, SAIL does not handle identifiable data. The mechanism of data provision to SAIL will be described here, including briefly covering our compliance with legislative and regulatory requirements. With support from the SAIL technical team, data providers (e.g. General Practitioners, hospital, government departments, etc.) separate their dataset into two parts: a demographic component (name, address, date of birth, gender, NHS number) and a content component (such as medication, diagnoses, educational attainment). The content is sent directly to SAIL. The demographics are sent to NWIS, acting as a TTP to process the data on behalf of the data controller (the provider). NWIS is a National Health Service (NHS) organisation and is authorised to hold the PID. The only information sent to NWIS is such that they already hold and it does not include the associated content data. Because of this, and the separation principle in that only the original data provider sees the identifiable data with the associated content, the CLDC is not contravened. NWIS matches the records against the WDS database and replaces the demographics with an Anonymous Linking Field (ALF), based on an encryption of the NHS number. Only the ALFs with some minimal demographic data (including gender, week of birth and area of residence to 1500 head of population) are sent to SAIL for recombination with the content data. Because of this, SAIL does not become a data controller for PID, since we do not have access to, or control over, the data.

Because of the breadth of data held by SAIL, the databank cannot be considered anonymous in totality. This is not due to a risk of process reversal to reveal identity, since encryption precludes this taking place by either SAIL or NWIS. Instead it is one of attribute re identification (jigsaw attack), whereby combinations of variables could be used towards re-identifying individuals. As such, it is a feature of all pseudonymised and anonymised row-level datasets which retain high utility. For data processing to prepare anonymised data for researchers, we rely on the GDPR (Articles 6 and 9) provisions for a task carried out in the public interest [11]. We arrived at this position through a review of our policies and processes, in relation to the law and with reference to relevant legal cases. This research was led by a solicitor and it included preparing a brief for review and opinion by a QC specialising in information law.

We arrived at our position in relation to individual consent through consultation with the Research Ethics Service [15] and Health & Care Research Wales. SAIL is not required to seek additional consent to incorporate datasets arising from routine public service delivery. This is because it is not a research activity per se and data accessed by researchers are in anonymised form. In accordance with the GDPR, we provide privacy notices on behalf of data providers (in places such as in General Practice surgeries). These inform members of the public of data use, and individuals are able to opt-out of their data being provided to SAIL by informing their GP. The opt-out is enacted between the data provider and NWIS: in practice we have had less than 0.025% of the population make this request to date.

SAIL has a longstanding Consumer Panel comprised of members of the general public [16]. We established the Panel in 2011 based on consultations to learn from others who had set up similar structures, such as the Western Australia Data Linkage Branch [8]. From this, and through developments over the course of time, the Panel provides SAIL with advice on data protection issues from the perspective of service users and carers. We encourage researchers to meet with the Panel to discuss proposals being developed and receive a public viewpoint. The Panel is also represented on an independent Information Governance Review Panel (IGRP) to ensure that all proposals to use SAIL data receive a general public opinion. The IGRP is described in a later section.

### Privacy-by-design

Privacy-by-design is an important concept to ensure that an appropriate set of control measures is built in and applied at all stages of operations, rather than bolt on solutions [17]. However, that is not to say that a privacy-by-design system is rigid once created, as controls need to be robust, yet dynamic and upgradable. The SAIL privacy-by-design model encompasses a suite of physical, technical and procedural controls applied to the data and the data environment. It is evident from the literature that there are limits on the effectiveness of controls that can be applied to datasets to mitigate the risk of disclosure without compromising data utility. A detailed discussion of this issue is beyond the scope of this paper, but there is a wealth of literature on the failure of anonymisation and the privacy risks in purportedly anonymised data through attribute re-identification [18,19]. Because at the time precedent was even more limited, the ways in which we arrived at the SAIL privacy-by-design model included combinations of activities including: consultations with data providers, the public and other stakeholders, and iterative developments, refinement and improvement.

SAIL data are managed within a dedicated building, which was built to house our population data science initiatives. It is replete with a set of secure physical safeguards, including building-level access control, and limits on access to floors and zones within the building, with particularly strict controls on who has access to areas where data are prepared and loaded into SAIL. Technical controls include system-wide security, and tiered measures applied to control data access by researchers and monitor conduct with the data. These include the use of 2-factor authentication to confirm users’ identities through the use of Yubikeys [20], detailed system logging and query auditing, regardless of where the data user is based. SAIL uses an array of procedural controls including: data sharing agreements with NWIS and with data providers so that responsibilities and expectations are clear, and data access agreements signed by each researcher to set out the requirements for conduct with the data. Breaches of this agreement are dealt with via standard disciplinary channels. Researchers are subject to an organisational affiliation check and are required to complete an accredited Safe Researcher training module to work with SAIL data. All proposals to use SAIL data are put to the IGRP, from whom a favourable opinion is required before data access can be granted.

In terms of controls applied to the data, we operate mechanisms from the whole system level down to the individual project level. We re-encrypt the ALFs following their receipt from NWIS, so that it is not possible for either a SAIL researcher or someone working for NWIS to use SAIL data to reverse the process to reveal NHS numbers. We also mask practitioner codes to protect professional reputations and avoid performance management. In preparing project data for access by researchers, we are able to apply a range of controls and to tailor these to protect the data and retain maximal utility. This includes encrypting all real world codes or anything that might lead to identification as well as re-encrypting the ALF again so that researchers running more than one project cannot use this key to link together datasets from discrete studies. We also apply data minimisation techniques such as aggregation (e.g. age to bands) and suppression of variables or entire records if deemed risky and/or not required to answer the research question. After data users have completed their analysis, their proposed outputs are scrutinised for disclosure risk by a SAIL senior analyst so that results (typically, statistical coefficients, tabulations and graphs, not row-level data) can be exported from the system.

We have endeavoured to work closely with data providers, the public and other stakeholders from the outset, as their satisfaction with SAIL is paramount. Since the operation of SAIL spans all stages from sourcing datasets of interest through to data archiving, it was essential that we were able to design a system to safeguard the data, individuals and providers at all points; not merely to comply with the law, but to engender and maintain good relationships for ethical conduct and integrity. As further evidence and assurance of security, we have also been successful in gaining ISO27001 certification for the SAIL databank, as well as for the UK SeRP platform.

### Data linkage

Linkage of SAIL datasets is by means of the ALF: the unique, anonymous identifier assigned by NWIS to each person represented in a dataset following a matching process against the WDS database. The matching algorithm was co-designed and tested by NWIS and SAIL. It operates at NWIS, and it is an automated ‘black box’ system, i.e. no one sees the identifiable data being processed. Only brief details are given here as the work is described fully in another publication [21]. We firstly set out to assess the suitability of using the NHS number as the basis of a unique identifier, and then to test the algorithm to match a selection of datasets from primary care, secondary care and social services against the WDS. The algorithm begins by comparing NHS numbers between the received dataset and the WDS, and then moves on to further variables: first name, surname, date of birth, gender and postcode. A UK postcode covers 15 properties on average but varies by population density [22]. Through the process of development, we were able to refine and apply the algorithm to provide consistently high matching accuracy [21]. A further advantage in NWIS carrying out the matching process is that ALFs can still be allocated to datasets deriving from outside the health sector (such as social services, or education) or where the NHS number is otherwise absent. In these cases, the matching process relies on the further variables and then the NHS number recorded in the WDS database is allocated to the record. NWIS maintains the WDS database as part of its service in providing national data and statistical information for NHS Wales. Each time a data provider sends a data extract destined for SAIL to NWIS, it is matched against the WDS and thresholds of match accuracy are sent to SAIL along with the ALFs and the minimal demographics. In addition to ALFs, we also have a similar process for allocating unique linkage keys to residential properties based on postal addresses. Through the use of these Residential ALFs (RALFs), researchers are able to associate individuals within the same home, and carry out health geographic studies without knowing the actual location of the properties in space [23].

### Data sources

SAIL holds a wealth of Wales-wide datasets, designated as core or core-restricted. The distinction is that data providers reserve the right to review proposed uses of core-restricted datasets, in addition to IGRP approval, whereas the use of core datasets can be approved by the IGRP alone. This is managed by SAIL staff concurrently with the IGRP process. The current core and core-restricted datasets are listed in Appendix 1 with further information available on the SAIL website [24]. Datasets are updated regularly with the periodicity varying between data providers. In addition, SAIL is able to incorporate study datasets collected by researchers, provided that they have obtained all relevant regulatory approvals, including consent if applicable, to allow them to transfer the dataset to SAIL and link it to existing SAIL data. Depending on the regulatory approvals, access to a study dataset might be limited to a particular project team or an individual researcher.

### Data Access

Access to SAIL data is dependent on a favourable opinion from the IGRP. This is an independent panel comprising representatives of professional bodies (such as Public Health Wales), of regulatory bodies (Research Ethics Service), and members of the public drawn from the Consumer Panel. It was constituted following consultation with data guardians of major Welsh datasets. The IGRP assesses the suitability of each proposal in terms of public interest and sensitivity risk [6,7]. Following their review, proposals may be approved directly, require amendment before they can proceed, or be refused if the study plan cannot be revised sufficiently to mitigate concerns. Although ethical approval is not required for the use of anonymised data in the UK, having an ethics committee representative on the panel is an additional safeguard as it means that all proposals are checked. As noted, SAIL also incorporates datasets collected for research, and in these cases, the researcher may be required to seek ethical approval. It is valuable to us to receive independent opinion on the use of the data in our aim to be objective and transparent.

Individuals applying for data access are subject to an identity check to ensure their credibility and organisational affiliation. Researchers from the public sector (such as higher education, the health service, social service, local or national government, charities) are allowed to access data following all necessary approvals. Researchers from the private sector are not allowed direct access to data, but are required to collaborate directly with SAIL or another public sector organisation [7]. Following IGRP and any additional approvals required, an anonymised project dataset is prepared for the researcher by a SAIL analyst. SAIL data can be accessed from anywhere a researcher is based through the use of remote desktop protocol so that analysis can be carried out in the UK SeRP environment, sometimes referred to as the SAIL Gateway [7]. Access within this virtual environment, surrounded by the array of physical, technical and procedural controls is the standard operating model of SAIL, with results, not row-level data released externally. This model has proved effective and serves us well in enabling data access and safeguarding the data. However, there are occasional cases where a release model is used. For example, we have provided row-level datasets to other accredited systems such as UK Biobank [25]. But crucially, this only occurs where the reputation of the recipient is assured and all relevant approvals, including explicit participant consent, are in place.

### Noteworthy outputs

SAIL is designed to maximise opportunities to conduct longitudinal, cross-sectoral evaluations of services, interventions and strategies whilst still protecting privacy. It has enabled large-scale evaluations of the impact of health and social exposures, natural experiments and policy interventions to be undertaken, involving billions of records and calculations. These include development of total or segmented population e-cohorts, embedded trials and studies into health and educational impacts of natural experiments, including changing density of alcohol outlets, targeted area regeneration, free school breakfasts, living with mental health and alcohol problems, and the impact of large-scale housing improvements [26-31]. SAIL has over 1,200 registered data users, and the data have been used in over 300 projects, with approximately 30 new projects each year. Three varied illustrations are given below.

Research using SAIL has been used to inform National Institute for Health and Care Excellence (NICE) guidelines and European Guidelines for the care of patients with ankylosing spondylitis (AS). Previously, disease-modifying drugs could only be offered to patients having severe disease for at least 3 months, in case the exacerbation was a flare rather than sustained severe disease. However, the work with SAIL showed that a flare lasts at most 1 month and so concluded that there is no reason to delay treatment for people beyond this duration, leading to improved options for AS care. It has also provided estimates of the annual cost of treating AS in the UK to inform service planning [32-33].

The Welsh Electronic Cohort for Children (WECC) includes anonymised health records of the >800,000 children living in Wales between 1990 and 2008 plus education records from 1994 onwards. This complex cohort enables numerous research questions to be addressed. For example, WECC has revealed factors that increase the likelihood of respiratory admissions up to age 5 years; and that children who move house frequently have an increased risk of poor health and educational underachievement [34-36].

According to Alzheimer UK, 90% of people with dementia experience behavioural and psychological symptoms and that, as a result, they might be prescribed antipsychotic drugs. However, there is a growing concern about the inappropriate use of antipsychotics in the elderly. Using SAIL data, increased risks of serious adverse medical outcomes in older people with dementia who are exposed to antipsychotic medication were demonstrated. These results are supporting recommendations for reducing the use of antipsychotic drugs for people with dementia, which is a key element of the dementia plan for Wales and a national priority in England [37].

## Discussion

The SAIL Databank has been operational since 2007 and has become an internationally-recognised centre of excellence for the its safe provision of anonymised datasets for research. It has received considerable acclaim, such as from the Organisation for Economic Co-operation and Development (OECD) and the Council of Canadian Academies [38-40]. However, we must be cognisant of change in this rapidly evolving area, and to be responsive and dynamic, so we continually learn and improve.

### Lessons learned

We outline some of the significant lessons we have experienced, and the application of the learning, for continual improvement in running the SAIL Databank:

- **Lesson:** Legislative and regulatory frameworks for data protection and the conduct of research change from time to time, for example, the enactment of the GDPR in 2018. Such changes can present a challenge in themselves, such as needing to ensure an appropriate lawful basis for data processing under new legal provisions, but the ways in which frameworks may be interpreted by gatekeepers carry their own challenges and can hinder research [41].
- **Application:** We carried out a piece of legal research and consulted a QC for advice on our lawful position under the GDPR. We did not need to make material changes to the operation of SAIL, but we did need to update our policies to reflect the new provision. We have been able to use this information when responding to queries from data providers to support them in their due diligence processes for the lawful provision of data in compliance with the GDPR.
- **Lesson:** Technological advances have the potential for greater safeguards but also greater threats. There is an array of hardware and software solutions to protect, manage and provide safe access to data in a secure environment. But, it is well-known that systems are subject to multiple external threats, such as from hacks, trojans, viruses or malware [42].
- **Application:** We have designed the SAIL Databank to be surrounded by multiple, tiered security mechanisms. These include: monitoring internet traffic so that attack attempts can be blocked; blocking suspect IP addresses and web domains; multi-vender internal & perimeter firewalls; network segmentation; weekly automated penetration & vulnerability scanning; automated patching and antivirus; secured infrastructure wide system logging; closed circuit television (CCTV); and delegated management of building access control; as well as regularly and reviewing and updating our security protocols.
- **Lesson:** The expansion of existing data and the growing availability of new and emerging data types (such as genomic, free-text clinical data and non-health administrative data) open up enhanced opportunities for research but, along with other commitments, require increases in analytical capacity and skills on limited budgets.
- **Application:** We have expanded the skill base amongst our analysts by means of PhD studentships, self-directed and team-based learning, and time-limited project investments through a variety of funders. However, with the demands on SAIL data, this remains a perennial problem, requiring further investment for expansion sustainability.
- **Lesson:** Societal views on data sharing are complex, evolving and depend on many factors, such as personal preferences, cultural values and the influence of mass media [43]. Public trust is paramount if debacles such as care.data, where insufficient information was provided, are to be avoided [44,45]. Even so, it is not enough merely to convey information, but to engage in an ongoing public dialogue, since demonstrating trustworthiness is necessary to engender trust.
- **Application:** We have a programme of public involvement and engagement (PI/E) including: a Consumer Panel to advise on our work; research studies focused on public engagement to assess public views and gain input; surgeries to guide researchers on including PI/E; and a series of public-facing research case studies. This programme is tailored to meet the needs of the public in varying contexts and settings for effective two-way communication.

### Further work

The work of SAIL is ongoing across all aspects of its operation as we strive for continual improvement in service provision and research outputs. We provide examples of challenges from three work areas, noting that it is beyond the scope of this paper to provide anything other than a brief description here.

#### New and emerging data types

The incorporation of emerging data types brings particular challenges, which we illustrate in relation to genomic and free-text data. Unlike structured microdata, where specified types of variable are entered in data fields, native genomic and free-text data exist in different formats. Genomic data can range widely from sequence to presence / absence of a genetic trait. Variant call format (VCF) files are text files describing the position, number and type of variants are valuable for research alongside structured phenotypic and lifestyle data. Free-text data are recorded in the course of public service and care provision, and include examples such as clinic letters and practitioners’ notes. What these data types have in common is that they can be more difficult to de-identify reliably than coded data [46-48]. We have teams of researchers working on methodologies, data governance and social acceptability to create solutions for SAIL.

#### Alternative means to enable data access

Although SAIL operates as a data repository, some data cannot be provided to the databank via our standard mechanisms using the separation principles and the use of a TTP to handle the PID. This can occur when there is no legal gateway for the movement of the PID pertaining to certain datasets from government departments, or when research access to data held at another data centre is required in conjunction with SAIL data. In these cases, we need to find alternative means to enable data access. We are using two approaches to address this issue. One involves using methodologies for privacy-preserving record linkage (PPRL) developed at Curtin University (Western Australia) based on Bloom filters [49,50]. In this way, only one-way hashed PID would be moved. The other involves the development of federated data access, rather than moving the data. A variety of options is being explored including inter-centre connections to allow a researcher to access data held at multiple sites, or to enable queries to be applied to distributed data [51].

#### Efficiency and sustainability

Budget constants and rapid multi-faceted developments in the data and societal landscapes are constant challenges and require ongoing effort and adaptation. These are embodied in issues such as how to: be efficient in meeting the requirements of multiple programmes and funders; enable safe access to highly granular data to maximise research potential; and meet the needs of varied research communities. We endeavour to take a ‘do once, use many times’ approach, as per the flexible, multi-functional UK SeRP and NRDA developments. This is a pattern we follow across the other centres we host so that we don’t work in silos but share learning and expertise to avoid duplication of effort. We also highly value opportunities for collaboration and mutual learning such as those afforded by the International Population Data Linkage Network (IPDLN).

## Conclusions

SAIL provides a working example of an extensive, whole population data resource for research. The data are being used widely in a range of studies focusing on determinants of health and well-being. Its privacy-by-design model and UK SeRP technology have received international acclaim, and we continually endeavour to demonstrate trustworthiness to support data provider assurance and public acceptability in data use. Further developments are underway to open up new opportunities and to streamline our processes. We strive for further improvement and continue a mutual learning process with our contemporaries in this rapidly developing field.

## Appendix 1. SAIL core and core-restricted datasets

The following is a summary of the core and core-restricted datasets held in the SAIL Databank. Further information can be found at: https://saildatabank.com/saildata/sail-datasets/

The information shown was retrieved on 23 May 2019.

**Table:**

Core datasets	Features (with approximate volumes)

Annual District Birth Extract	Office for National Statistics (ONS) register of all births in Wales collected from birth registrations. 35,000 births per year, from 2003 to present.
Annual District Death Extract	ONS register of all deaths relating to Welsh residents, including those that died out of Wales, collected from death registrations. 32,000 births per year, from 2003 to present.
Critical Care Dataset	Nation-wide critical care database to monitor quality of service, to drive improvements and policy development, collected and coded at each hospital. 9,000 admissions per year, from April 2006 to May 2016.
Diagnostic and Therapy Services Waiting Times	Waiting times for specified diagnostic and therapy services for the NHS in Wales, submitted by Local Health Boards. 120,000 records per year from October 2005 to May 2016.
Emergency Department	Administrative and clinical information for all NHS Wales Accident and Emergency department attendances, collected and coded at each hospital. 750,000 attendances per year from 2009 to present.
National Community Child Health Database	Birth registration and monitoring of child health examinations and immunisations, bringing together data from local Child Health System databases, held by Local Health Boards. 35,000 births and 500,000 vaccinations per year, from 1987 to present.
Outpatient Dataset	Attendance information for all NHS Wales hospital outpatient appointments, collected from the central PAS (Patient Administrative System). 4,500,000 appointments per year from 2004 to present.
Outpatient Referral	Referral pathways to secondary care, submitted by Local Health Boards. 1,000,000 records per year, from 2009 to Jun 2016.
Patient Episode Database for Wales	NHS Wales hospital admissions comprising attendance and clinical information, diagnoses and operations performed, collected from the central PAS (Patient Administrative System). 950,000 hospital admissions per year, from 1997 to present.
Postponed Admitted Procedures	Information on reason for cancelled admitted procedures, submitted by Local Health Boards. 80,000 records per year, from 2013 to May 2016.
Primary Care GP dataset	Signs, symptoms, test results, diagnoses, prescribed treatment, referrals for specialist treatment and social aspects relating to the patient’s home environment, collected from General Practices. Averaging around 5000 patients per practice, from between January 2000 to October 2014 (depending on practice) to present.
Referral to Treatment Times	Monitoring the 26-week referral to treatment time target, submitted by Local Health Boards. 450,000 records per year, from September 2011 to May 2016.
UK Health Dimensions	NHS related lookup tables, including version 2 and version 3 Read codes, ICD10 codes, cross-coding system mappings, organisational information, and geographic information, from various reference data providers. Comprises thousands of reference tables.
Welsh Demographic Service Dataset	Administrative information about individuals in Wales that use NHS services, drawn from GP practices. Current and past population of Wales, from 1990 to present (approximately 5 million people).

**Table:**

Core-restricted datasets	Features (with approximate volumes)

Active Adult Survey	Large scale survey of the adult population in Wales covering sport related activities, provided by Sport Wales. Data on 13,000 individuals between January 2012 and January 2013.
Bowel Screening Wales	Administrative and clinical information for bowel screening, collected by Public Health Wales. 140,000 screening test records per year, from 2008 onwards.
Breast Test Wales	Administrative and clinical information for breast screening, collected by Public Health Wales. 110,000 screening test records per year, from 1989 onwards.
Cervical Screening Wales	Administrative and clinical information for cervical screening, collected by Public Health Wales. 225,000 screening test records per year, from 1990 onwards.
Congenital Anomaly Register and Information Service (CARIS)	Information about foetuses or babies who has or is suspected of having a congenital anomaly, collected by CARIS. 1,500 babies per year, from 1998 to 2011.
Educational Attainment	Annual return submitted by all sectors including nursery, primary, middle, secondary and special schools, provided by Welsh Government. 470,000 records per year, from 2003 to 2015.
National Survey for Wales	The Welsh Government’s major survey of the general population in Wales covering a wide range of topics affecting people and their local area. 12,000 people per year, from 2012 to 2015.
Welsh Cancer Intelligence and Surveillance Unit (WCISU)	National Cancer Registry for Wales to record, store and report on all incidences of cancer, collected by WCISU. 686,000 records, from 1972 to present.
Welsh Health Survey	Information on the health and health-related lifestyles of people living in Wales, collected by the National Centre for Social Research. 4,400 records from April 2013 to December 2014.

## Abbreviations

Common Law Duty of Confidentiality (CLDC); Economic and Social Research Council (ESRC); General Data Protection Regulation (GDPR); Health and Care Research Wales (HCRW); Health Data Research UK (HDRUK); Medical Research Council (MRC); National Research Data Appliance (NRDA); NHS Wales Informatics Service (NWIS); Person-Identifiable Data (PID); Secure Anonymised Information Linkage (SAIL); Trusted Third Party (TTP); United Kingdom Secure Research Platform (UK SeRP); Welsh Demographic Service (WDS)
